# Contemporary insights into the pathogenesis, diagnosis and therapy of pulmonary arterial hypertension

**DOI:** 10.5830/CVJA-2010-088

**Published:** 2010-12

**Authors:** Mohammed R Essop

**Affiliations:** Division of Cardiology, Baragwanath Hospital and University of the Witwatersrand, Johannesburg, South Africa, and Chairman, Pulmonary Hypertension Interest Group

**Keywords:** pulmonary hypertension, mechanisms, therapy

## Abstract

**Summary:**

Current data challenge the concept that pulmonary arterial hypertension (PAH) is purely a disorder of impaired vasomotor tone. Instead, we recognise today that the phenotype of PAH represents the complex and disordered regulation of expression of key signalling molecules and abnormal molecular trafficking. Discovery of mutations of the ubiquitous receptors of the transforming growth factor beta (TGF-β) superfamily in many patients with PAH has been instrumental in unravelling the pathobiology of this otherwise fatal disorder. Much still needs to be learnt before we are able to substantially alter the natural history of PAH. Until such time, therapies that fundamentally attempt to restore vasomotor tone continue to be developed and tested.

Current clinical research in the therapeutic arena is focused on defining the best permutation of the three major groups of drugs – prostacyclin analogues, phosphodiesterase type-five inhibitors and the endothelin receptor antagonists. However, if we are to make any significant impact on the otherwise dismal outcome of PAH, we have to recognise that even more important than the challenge of new therapies, is the challenge in diagnosing the condition early in the course of its relentless progression to right heart failure and eventual death.

## Summary

Since the first recorded description of pulmonary arterial hypertension by Romberg in 1891,^1^ a series of landmark observations have culminated in a deeper understanding of the pathobiology, diagnosis and therapy of the disease as we know it today. This review, compiled by a search strategy of PubMed, using the terms pulmonary arterial hypertension with specification for articles published in English, seeks to provide contemporary and concise answers to the specific questions posed and concentrates specifically on category 1 (see later) pulmonary hypertension (PH) under the rubric of pulmonary arterial hypertension (PAH). For a more general overview, the reader is referred to several excellent recently published documents on guidelines, classification and therapy of PH.^2^-^5^

An increased awareness of PH in South Africa is particularly important for several reasons, all of which have served as an impetus for the formation of the Pulmonary Hypertension Interest Group. Firstly, there is reason to believe that PH in general is a prevalent condition in this country. Apart from the frequent contribution of valvular heart disease, cardiomyopathy and congenital heart disease, missed at the time of birth and during infancy, to the overall burden of PH, preliminary data from elsewhere suggest that retroviral infection may now be the leading cause. The incidence of PAH in cohorts of patients with HIV is estimated at about 0.5%.^4^ Even with a conservative prevalence of five million people infected with HIV in South Africa, this would translate to some 25 000 patients with PAH. This complication is rarely diagnosed, unfortunately carries with it a poor prognosis independent of the CD_4_ count or viral load, and does not appear to be responsive to highly active anti-retroviral therapy. Secondly, as in many countries elsewhere, an advocacy group is sorely required here to promote all aspects of the diagnosis and therapy of PAH and importantly, to lobby funders not to shirk their responsibility toward the management of this small but desperate group of patients.

## What are the pathological hallmarks of PAH?

PAH is a disease of the pre-capillary pulmonary arterial bed, including the medium-sized pulmonary arteries and pulmonary arterioles characterised by vascular obliteration. Current knowledge implicates unchecked proliferation of smooth muscle cells and dysregulated control of endothelial cells with apoptosis and dysfunction in some areas and profuse proliferation in others.

**Fig. 1.**
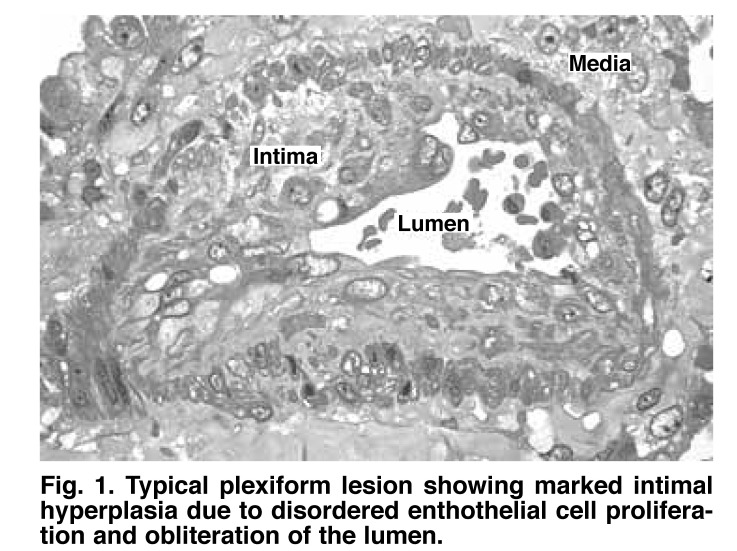
Typical plexiform lesion showing marked intimal hyperplasia due to disordered enthothelial cell proliferation and obliteration of the lumen.

Plexiform arteriopathy (Fig. 1) is the pathological hallmark of advanced PAH and represents a chaotic assembly of proliferating endothelial cells, smooth muscle cells, fibroblasts and possibly circulating and bone marrow-derived endothelial progenitor cells. Proximal to these lesions, the pulmonary arterioles are dilated, have a paucity of endothelial lining, and show proliferative smooth muscle cells, fibroblasts and adventitia. Whereas distal loss of vasculature was previously thought to be secondary to more proximal obstruction, compelling recent evidence suggests that this may be an active process linked to increased apoptosis of endothelial cells and pericytes.^5^

While pulmonary veno-occlusive disease and pulmonary capillary haemangiomatosis share some features in common with other causes of PAH, they are pathologically distinct and have been included in a separate category termed 1′ by the European Society of Cardiology (ESC) and the European Respiratory Society (ERS) guidelines.^3^

## Is there a unifying pathogenetic mechanism for PAH?

**Fig. 2.**
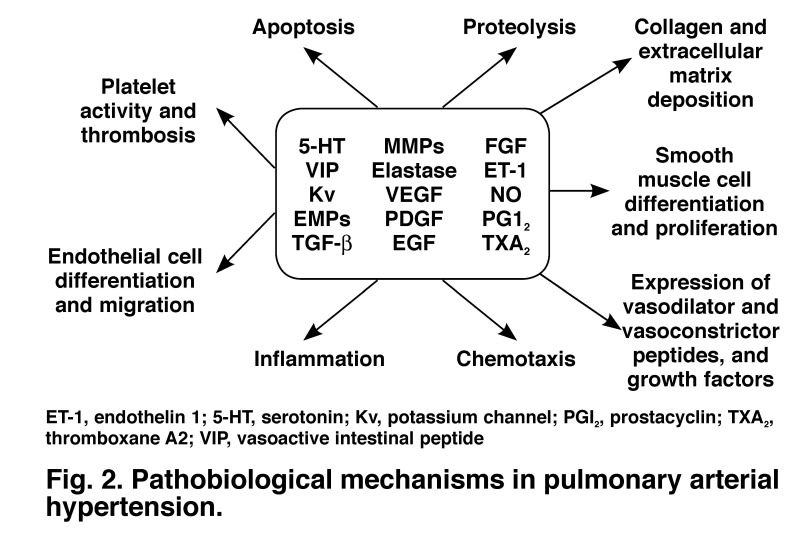
Pathobiological mechanisms in pulmonary arterial hypertension.

The complexity of receptor activation, signalling molecules and downstream pathways with cross-talk at different levels between these makes it unfortunately difficult to pinpoint a precise mechanism for PAH (Fig. 2). Nevertheless, the discovery of a loss-of-function mutation in bone morphogenetic receptor II (BMPR2), a member of the TGF-β superfamily, in 20 to 30% of patients with idiopathic PAH (IPAH) and 60% of patients with familial PAH immediately paved the way for elucidating the pathobiology of the disease.^6^,^7^

TGF-β receptors together with their ligands (proteins that bind to the receptor) control diverse processes involved in vascular remodelling, including among others, cell proliferation and apoptosis, cellular differentiation, and collagen and extracellular matrix turnover. These are all processes that are fundamentally involved in PAH but the precise link between the genotype and the expression of the pulmonary hypertensive phenotype remain elusive.

**Fig. 3.**
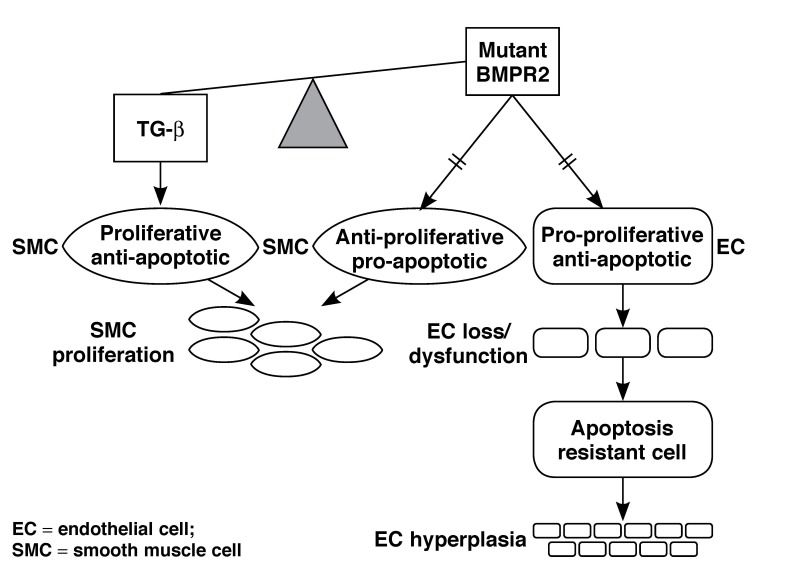
Unifying mechanism for smooth muscle cell proliferation, endothelial loss and proliferation in patients with loss-of-function mutation in BMPR2.

According to the current hypothesis, a loss-of-function mutation in the BMPR2 receptor results in an imbalance in the equilibrium between the opposing effects of TGF-β and BMP signalling, which in the smooth muscle cell (SMC) favours a pro-proliferative and anti-apoptotic response but in the case of the endothelial cell (EC), has an anti-proliferative and pro-apoptotic effect (Fig. 3). Although the reason for the contrasting effect of BMP signalling on the SMC and EC remain unknown, the model provides compelling evidence for the pathobiological underpinnings of PAH. Furthermore, emergence of apoptosis-resistant clones of ECs may account for unregulated proliferation and the formation of plexogenic lesions.^8^

## How is PH best defined, classified and investigated?

Although PAH is optimally defined on pathology, this is rarely possible except post-mortem. From a haemodynamic perspective, pulmonary vascular resistance is the best measure of the resistance of the pulmonary circulation to flow but this in general requires invasive cardiac catheterisation, which is inappropriate as a screening test. Therefore an estimate of pulmonary artery pressure forms the starting point for diagnosis of PH.

The time-honoured clinical methods, including a left parasternal heave, palpable P2, pulmonary ejection click and narrow A2-P2 split are important, as are the ECG showing P-pulmonale, right-axis deviation and a tall R in V1 and chest X-ray with characteristic dilatation of the proximal pulmonary arteries and attenuation of distal third of the vasculature. However, echo-Doppler is an easily available, inexpensive and non-invasive way of obtaining a comprehensive assessment of pulmonary haemodynamics and is recommended as the initial investigation of choice in most guidelines.^2^,^3^

Apart from measuring pulmonary artery systolic and diastolic pressure from the tricuspid and pulmonary regurgitant jets, respectively, echocardiography allows exclusion of left heart pathology and congenital shunts as possible aetiologies. Additionally, right ventricular size and function, which have important prognostic value, may be derived using a variety of indices. The echocardiographic definition of PH is a pulmonary artery systolic pressure greater than 40 mmHg.

**Fig. 4.**
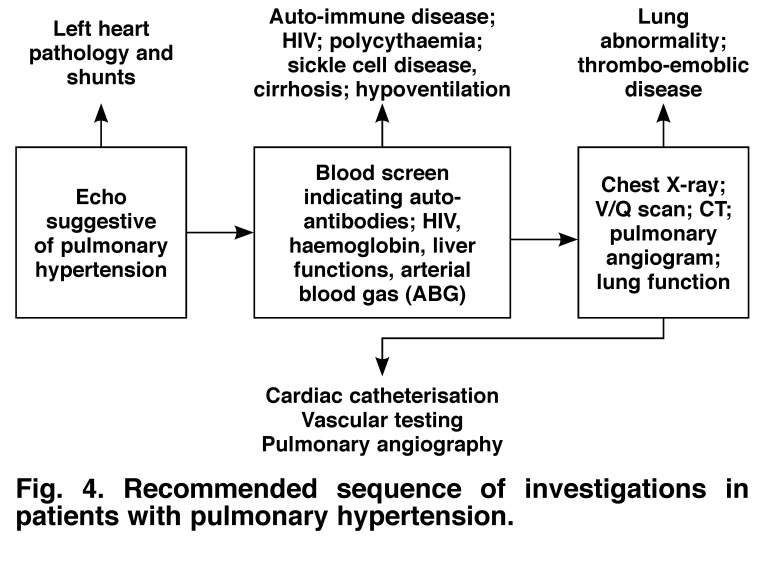
Recommended sequence of investigations in patients with pulmonary hypertension.

Once suspected, a series of investigations should be undertaken (Fig. 4) in order to define the cause of PH. The sequence of testing indicated in the algorithm serves merely as a guideline – investigations should be guided by the most cost-effective approach and based on sound clinical judgment.

The classification of PH has undergone a series of modifications since the original description endorsed by the WHO in 1973, with the latest proposal published from a symposium held in Dana Point, California.^4^ The classification essentially seeks to create categories of PH that share pathological and clinical features and have similar therapeutic options and is summarised in Table 1.

**Table 1.** Updated Clinical Classification Of Pulmonary Hypertension (Dana Point, 2008)

1 Pulmonary arterial hypertension (PAH)
1.1 Idiopathic
1.2 Heritable
1.3 Drugs and toxin induced
1.4 Associated with (APAH)
1.41 Connective tissue disease
1.42 HIV infection
1.43 Portal hypertension
1.44 Congenital heart disease
1.45 Schistosomiasis
1.46 Chronic haemolytic anaemia
1′ Pulmonary veno-occlusive disease and/or pulmonary capillary haemagiomatosis
2 Pulmonary hypertension due to left heart disease
2.1 Systolic dysfunction
2.2 Diastolic dysfunction
2.3 Valvular disease
3 Pulmonary hypertension secondary to lung disease and/or hypoxia
3.1 Chronic obstructive pulmonary disease
3.2 Interstitial lung disease
3.3 Lung diseases with mixed restrictive and obstructive patterns
3.4 Sleep-disordered breathing
3.5 Alveolar hypoventilation disorders
3.6 Chronic high-altitude exposure
3.7 Developmental abnormalities
4. Chronic thrombo-embolic pulmonary hypertension
5. Pulmonary hypertension with unclear and/or multifactorial mechanisms
5.1 Haematological disorders: myeloproliferative disorders, splenectomy
5.2 Systemic disorders: sarcoidosis, neurofibromatosis, vasculitis
5.3 Metabolic disorders: glycogen storage disease, Gauchers, thyroid disorders
5.4 Others: tumour obstruction, fibrosing mediastinitis, chronic renal failure on dialysis

## Is cardiac catheterisation indicated in every patient with suspected PAH?

Despite advances in non-invasive imaging, including echocardiography, CT and MRI, invasive left and right heart catheterisation is imperative to confirm pulmonary hypertension, rule out passive (category 2) aetiologies and intra-cardiac shunts, assess for acute vaso-reactivity and for prognostication purposes. Pulmonary angiography is not routinely necessary but may be useful to exclude thrombo-embolic disease, vasculitis and peripheral pulmonary artery stenosis.

The haemodynamic definition of PAH is a mean pulmonary artery pressure greater than 25 mmHg at rest or 30 mmHg with exercise, with a pulmonary capillary wedge pressure or left ventricular end-diastolic pressure less than 15 mmHg and a pulmonary vascular resistance greater than 3 ru. When there are marked respiratory variations in wedge pressure, values should be measured at end-expiration.

Oxygen and inhaled nitric oxide (10–30 ppm) are the most useful agents for acute vaso-reactivity testing in the catheterisation laboratory. Inhaled nitric oxide has the advantage that it acts selectively on the pulmonary circulation, since it is rapidly inactivated by haemoglobin on entry into the blood stream and it does not cause ventilation–perfusion mismatch because it is only distributed to lung segments that are normally ventilated.

Only 5 to 10% of patients subjected to vaso-reactivity testing are classified as responders, variously defined as an absolute reduction in mean pressure greater than 10 mmHg to a mean value less than 40 mmHg, or a greater-than 20% reduction in mean pressure and pulmonary vascular resistance.^9^

## What is optimal medical therapy for PAH?

General measures for patients with PAH include limitation of physical effort, vaccination against influenza and home oxygen for hypoxic individuals. Although not supported by rigorous evidence, prophylactic warfarin anticoagulation, diuretics for those with systemic venous congestion and digitalis for right ventricular dysfunction may be appropriate. Women of child-bearing age should be strongly discouraged from falling pregnant since maternal mortality is in excess of 50%, and should utilise appropriate techniques for contraception.

Patients identified as acute responders on vaso-reactivity testing benefit from high-dose dihydropyridine calcium antagonists but should be monitored closely since about one-half may relapse in the long term. Empirical use of calcium antagonists without vaso-reactivity testing, a practice that is not infrequent, should be strongly discouraged.

Current therapy specifically targeted at PAH comprises the prostanoids, endothelin antagonists and phosphodiesterase type-5 inhibitors. The prostanoids may be administered intravenously (epoprostenol, trepostinil and iloprost), subcutaneously (trepostinil), by inhalation (iloprost) or orally (beraprost). Endothelin antagonists may cause non-specific blockade of both the type A and B receptor (bosentan) or selective inhibition of the type A receptor (sitexantan and ambrisentan). The phosphodiesterase type-5 inhibitors (sildenafil, revatio and tadalafil) increase nitric oxide availability by inhibiting the breakdown of the second messenger, cyclic guanosine monophosphate. For a more detailed account of these drugs the reader is referred to a recent review,^10^ but unfortunately, apart from the phosphodiesterase type-5 inhibitors, none of these therapies are available in this country.

Based on published evidence, all class 4 patients should receive intravenous epoprostenol, since this is the only therapy that has demonstrated a survival benefit in patients with PAH.^10^ The choice of therapy in class 2 to 3 patients and the optimum combination of drugs are as yet unclear. Also unknown is whether therapy should be initiated in asymptomatic patients with confirmed PAH. Apart from epoprostenol in class 4 patients, importantly, no therapy has shown significant survival benefit and the nature and magnitude of an appropriate surrogate endpoint are still debatable.

Patients refractory to medical therapy and with advanced symptoms may be considered for balloon atrial septostomy or lung transplantation. The rationale of creating a right-to-left shunt percutaneously is based on the fact that patients with Eisenmengers syndrome have a much better survival than other groups of PAH, perhaps because of the possibility of unloading the right ventricle and increasing systemic blood flow, even though this may be at the expense of desaturated blood.

## Does the future hold any promise for new therapies for PAH?

The still-dismal outcome of patients with PAH has stimulated intense and exciting avenues of research into new therapies that would hopefully reverse the pulmonary hypertensive phenotype. As we delve deeper into the pathobiology of PAH, we increasingly realise the complexity of pathways involved in vascular remodelling. While many new approaches have shown promise at the bench side and in the monocrotaline-induced rat model of PAH, these remain to be tested in the clinical arena.

Inhibition of TGF-β receptors with antibodies or losartan, statins which increase BMPR2 gene promoter function, inhibition of tyrosine kinase, which is an important mediator of cellular proliferation and migration, with drugs such as imatinib, and alteration of intracellular signalling via the SMAD pathway are but a few examples of potential therapeutic targets. Since right ventricular function may be an even more robust predictor of outcomes than the actual level of pulmonary pressure, more detailed study of right ventricular remodelling and the molecular determinants of hypertrophy and failure of this chamber are also crucial.

## Conclusion

In the not-too-distant future, we may be able to provide our patients with PAH with a cure. Until such time, however, it behoves us as clinicians to always consider the possibility of PAH, institute a diligent workup to make a precise diagnosis and assessment of severity and reversibility, and do all we can to make available potential therapies to this desperate group of patients.
